# Strategic perspectives, creativity, and financial performance in Vietnamese SMEs

**DOI:** 10.1016/j.heliyon.2023.e20354

**Published:** 2023-09-21

**Authors:** Phuong Ngoc-Duy Nguyen, Khuong Ngoc Mai, Thu-Hang Le

**Affiliations:** a Vietnam National University, Ho Chi Minh City, Viet Nam; b School of Economics, Finance and Accounting, International University, Vietnam National University-HCMC, Viet Nam; c School of Business, International University, Vietnam National University-HCMC, Viet Nam

**Keywords:** Resource-based view, Dynamic capabilities view, Inside-out perspective, Outside-in perspective, Organizational creativity, Financial performance

## Abstract

This study examines the relationships between the inside-out and outside-in perspectives of strategists, organizational creativity, and financial performance in Vietnamese small and medium enterprises (SMEs). Based on the resource-based view and dynamic capabilities view, we collected cross-sectional data from 382 managers at all levels of Vietnamese SMEs. The study employed partial least squares structural equation modeling. The findings confirm that the inside-out perspective positively affects organizational creativity but has a negative impact on financial performance. Conversely, the outside-in perspective positively affects both organizational creativity and financial performance. Additionally, the study reveals that organizational creativity positively affects financial performance and acts as a mediator between both perspectives and financial performance. Thus, the study suggests that SMEs can benefit from adopting an outside-in perspective to enhance both their organizational creativity and financial performance. Overall, the study contributes to the literature on the cognitive school of strategic management in SMEs.

## 1 Introduction

Small and medium-sized enterprises (SMEs) are crucial in driving economic growth and development worldwide, contributing significantly to employment, economic output, and innovation. Effective strategic management is paramount for the success of any organization, particularly SMEs operating in emerging economies [1]. In today's volatile, uncertain, complex, and ambiguous (VUCA) environment, the main responsibility of senior executives is to identify and build sustainable competitive advantages that guide their organizations toward success. The high financial performance of the firm is contingent upon its ability to intersect three crucial domains: catering to customer needs, being beyond the capabilities of competitors, and being within the firm's capacities [2,3]. To achieve such advantages, executives must consider both internal firm-related factors and external market-related factors. In this endeavor, the mental models of senior executives play a vital role as they shape the organization's strategic decisions, culture, and performance metrics. An essential aspect of strategic management is the strategist's mental perspective, which can either be inside-out or outside-in. The inside-out perspective emphasizes the organization's internal strengths [4], while the outside-in perspective emphasizes external factors such as market trends and customer needs [5].

Previous studies conducted a comprehensive meta-analytic synthesis and examined the relationships between inside-out and outside-in orientations and innovation performance [6]. Additionally, the literature emphasizes the significance of organizational creativity and open innovation in enhancing SMEs' performance and international business competence, shedding light on the direct impact of organizational creativity on financial outcomes [7]. Despite their relevance in defining firm success in general and executive decision making in particular, strategists' perspectives have remained a largely unexplored area in business research [8,9]. [8] highlight that the limited exploration of intuition and creativity in decision-making within strategic contexts in the management literature offers a valuable opportunity to gain insights into how strategists' cognitive processes influence organizational outcomes, including financial performance. Moreover, although the resource-based view (RBV) asserts that valuable, scarce, unique, and irreplaceable resources form the basis of enterprise competitiveness and economic rent [10], there is a need for a more comprehensive understanding of SMEs' competitiveness in dynamic and fast-changing environments. This is where the dynamic capability view (DCV) comes into play, proposing at the construction, integration, and re-configuration of resources are essential for thriving in volatile environments [11,12]. Additionally, while the global innovation index report has repeatedly highlighted the challenges faced by emerging markets in generating creative results, such as in Southeast Asia and Vietnam [13], there is a lack of empirical evidence conclusively considering the role of organizational creativity in the relationship between strategist's perspective, and SME financial performance.

To fill the research gaps, this research is based on the integration of RBV and DCV to investigate how inside-out perspective and outside-in perspective of executives affect SME financial performance both directly and indirectly through SME creativity. In particular, this study tries to answer the following three questions:

1. What are the relationships between two strategists' perspectives, including inside-out and outside-in perspectives, on SME creativity and financial performance?
2. What is the relationship between SME creativity and financial performance?
3. Are the relationships between two strategists' perspectives and financial performance mediated by SME creativity?

The research contributions of this study are twofold. First, there is still a gap in understanding the mechanism that guides strategists in making informed decisions, especially in the context of Vietnam SMEs. This research will address this gap by pioneering the use of both inside-out and outside-in perspectives in a research model to examine the strategic management process. Specifically, the study explores the mediating effect of creativity on the relationship between the mental perspectives of strategists and financial performance. Second, this study will contribute to the field of strategic management by empirically validating the extension of the resource-based view argument through the dynamic capability perspective proposed by Refs. [14,15]. By examining how organizations can create valuable, rare, difficult to imitate, and imperfectly substitute resources, as well as generate creativity in dynamic environments post Covid-19.

The upcoming sections will begin by outlining our paper's background and research model, followed by the presentation of research hypotheses. We will then provide an overview of the methods utilized in this research, which include research design, data collection, variables, and measures. Subsequently, we will present the empirical results obtained from our study. Lastly, we will discuss the implications of our findings for researchers and strategists, provide concluding remarks, and highlight potential areas for future research and any associated limitations.

## 2 Literature review

### 2.1 Literature review and theoretical background

The concepts of inside-out and outside-in orientations highlight the varying emphasis placed on internal and external resources and capabilities as sources of competitive advantage. In a meta-analytic synthesis of 232 independent studies [6], examines the relationships between inside-out and outside-in orientations and innovation performance while considering the moderating effects of industry type, economic development, and cultural context. The findings provide insights into the relative importance of each orientation for innovation performance and its direct and indirect effects on overall firm performance. Additionally, the literature has delved into the impact of organizational creativity on financial performance, especially concerning SMEs. Another significant area explored in the literature pertains to the influence of organizational creativity on financial performance, particularly in the context of SMEs. Nevertheless, studies have indicated positive effect of organizational creativity on SME performance, suggesting that this intangible resource has the potential to directly enhance overall performance [7,16].

[17] explores three strategic thought streams, namely adaptive marketing capabilities, dynamic capabilities, and resource-advantage theory, which underscore the need for firms to continually renew themselves in today's dynamic and hypercompetitive global economy. These streams contribute to the ongoing debate about whether strategic focus should be oriented inward or outward and whether strategies should be static or dynamic. The RBV emphasizes that a company's unique and valuable internal resources and competencies are the primary sources of competitive advantage. The inside-out orientation highlights the importance of leveraging these internal resources to achieve sustained competitive advantage. However, this perspective has been criticized for neglecting the external market environment, where the outside-in orientation comes into play. The market resource-based view (MRBV) suggests that firms should consider customer and competitor insights to formulate effective strategies. To respond to the dynamic market environment, the dynamic capability (DC) perspective is incorporated, emphasizing a firm's ability to build, integrate, and reconfigure resources to adapt to environmental changes. Organizational creativity, which refers to generating and implementing novel and useful ideas, is particularly relevant in this context, as it can be fostered by development management capabilities that allow the firm to seize opportunities, reconfigure resources, and learn from past experiences.

The existing literature reveals a lack of comprehensive understanding regarding the complex interrelationships between two strategists' perspectives, organizational creativity, and financial performance. To address this gap, our study proposes a theoretical framework combining the DC and RBV to offer a more comprehensive and robust lens for examining these interconnections. Specifically, we aim to explore the mediating role of organizational creativity on the link between the two strategists' perspectives and financial striving to achieve and sustain a competitive advantage in the rapidly changing business landscape.

### 2.2 Hypotheses development

The DC and RBV are two widely used theoretical frameworks in strategic management, which provide a theoretical foundation for this study's hypotheses development.

#### 2.2.1 Inside-out perspective

The inside-out perspective of strategist's centers on the idea that a company's strategy should be built around its strengths rather than external opportunities [6,18]. Successful companies focus on building a strong resource base over time, which provides access to unfolding market opportunities in the medium and short term. Identifying the difficult-to-replicate capabilities and distinctive assets that a company should acquire or enhance is the foundation of strategy formulation. This requires substantial investment and a forward-looking approach and has a substantial impact on the culture and identity of the organization. Positioning in the market is crucial, but tactical and constrained by a resource-oriented strategy. The inside-out perspective emphasizes the challenging and time-consuming nature of developing distinctive competencies over tangible resources, thereby making them a more desirable source of competitive advantage. Nonetheless, these competencies may also become rigid, preventing businesses from gaining access to new opportunities [19]. The inside-out perspective encourages companies to first build on their unique competences and attempt to find or create a more suitable market instead of reactively adapting to the unpredictable whims of the current environment.

Empirical studies conducted in Chinese SMEs have shown that internal resources and capabilities positively influence innovation, which is a critical driver of organizational creativity [20,21]. This approach is linked to a firm's ability to create and deploy resources dynamically to achieve a competitive advantage, according to the DCs view. It emphasizes the strategic value of a firm's resources and capabilities as sources of sustained competitive advantage, according to the RBV view. Therefore, an inside-out perspective of a strategist will positively affect organizational creativity in Vietnamese SMEs.

H1 Inside-out perspective of strategist positively affects organizational creativity in Vietnamese SMEs.

Although the inside-out perspective of a strategist can boost organizational creativity, as in Ref. [22] study of 161 Taiwan-based SMEs, it may also hinder financial performance, particularly if the focus on internal resources and capabilities hinders the ability of the firm to adapt to changing market conditions and customer needs [23,24]. From the RBV perspective, a firm's resources and capabilities are only valuable if they contribute to financial performance. At the same time, the DCs view emphasizes the dynamic deployment of resources to achieve competitive advantage. Similarly, a narrow focus on internal factors may limit a firm's ability to identify and capitalize on external opportunities, ultimately affecting its financial performance [25]. Hence, we hypothesize that the inside-out perspective of a strategist will negatively affect financial performance in Vietnamese SMEs.

H2 Inside-out perspective of strategist negatively impacts on financial performance in Vietnamese SMEs.

#### 2.2.2 Outside-in perspective

The outside-in perspective of a strategist emphasizes the importance of considering the external environment when determining a firm's strategy [26,27]. Successful businesses are market-driven, and they place a premium on adapting to the market's opportunities and threats, with an emphasis on satisfying customer needs and meeting competitive challenges [28,29]. Executives who follow an outside-in approach to strategy development commence with an evaluation of the external environment to recognize promising market prospects. The positioning tactic entails identifying a market position that could confer bargaining power to the company while simultaneously thwarting competitors [30]. Advocates of this perspective emphasize the importance of understanding the needs, competencies, positions, and objectives of all significant market or industry participants. Despite the importance of a company's resources and activities, an outside-in strategist views them as potential obstacles to its ability to execute the optimal business strategy. Therefore, the outside-in perspective emphasizes developing a strategic perspective that emphasizes market positioning and comprehending and responding to external developments.

By involving customers, especially lead users, in creativity processes, outside-in-oriented SMEs increase their likelihood of market success, as customers frequently have more creative ideas than internal product developers [31]. Through an outside-in orientation, SMEs are able to think expansively and explore gaps in demand by focusing on customer needs rather than product development alone [23]. In addition, in accordance with the dynamic capabilities perspective, SMEs can effectively respond to market challenges and opportunities by integrating external knowledge and resources and staying mindful of market trends, thereby enhancing their creative capacity to develop novel solutions [24,25]. As a result, adopting an outside-in perspective will enhance organizational creativity in SMEs.

H3 Outside-in perspective of strategist positively affects organizational creativity in Vietnamese SMEs.

Outside-in-oriented companies benefit from multiple feedback loops and ongoing dialogue with relevant stakeholders, allowing them to abolish cognitive barriers and gain valuable insights from end-users' perspectives [32,33]. In addition, aligned with RBV view, SMEs can effectively leverage external resources to compensate for their limited internal resources and competencies, enabling them to develop new technologies and capitalize on market opportunities [18]. By integrating customer knowledge, businesses can achieve this through methods like conducting market research or engaging in collaborative product and technology development with customers. By leveraging external expertise and customer insights, SMEs can gain a deeper understanding of market needs and preferences, paving the way for strengthening their financial performance [6]. Therefore, we propose the following hypothesis:

H4 Outside-in perspective of strategist positively impacts on financial performance in Vietnamese SMEs.

Table 1 summarizes the main differences between the outside-in and inside-out perspectives.

**Table 1:** Inside-out versus outside-in perspective.

	**Inside-out perspective**	**Outside-in perspective**
Emphasis on	Resources over markets	Markets over resources
Orientation	Strength-driven (internal potential)	Opportunity-driven (external potential)
Starting point	Resource base & activity system	Market demand & industry structure
Fit through	Adaptation of environment	Adaptation to environment
Strategic focus	Attaining distinctive resources	Attaining advantageous position
Strategic moves	Building resource base	External positioning
Tactical moves	External positioning	Acquiring necessary resources
Competitive weapons	Superior resources and imitation barriers	Bargaining power & mobility barriers

The ability of a firm to generate and implement new ideas that bring value is known as organizational creativity [34]. This capability is considered a key driver of competitive advantage, as both the RBV and DCs perspectives emphasize [25]. Notably [35], stated that organizational creativity can positively impact a firm's financial performance. Studies conducted in Asia-based SMEs have also shown that innovation is linked to enhanced financial performance [36,37]. Therefore, it can be argued that Vietnamese SMEs that prioritize organizational creativity will likely improve their financial performance. In a world where innovation and adaptability are increasingly critical to survival, fostering organizational creativity can be the key to achieving sustained success.

H5 Organizational creativity positively affects financial performance in Vietnamese SMEs.

Using the segmentation approach [38], this study proposes two hypotheses regarding the mediating effects of organizational creativity based on the significant effects of two strategists' perspectives on organizational creativity [22,24] and the significant effect of organizational creativity on financial performance [36]. This research contributes to a greater understanding of the mechanisms underlying creativity within organizations by examining the interplay between strategist perspectives and organizational creativity, as well as the latter's effect on financial performance.

H6 Organizational creativity mediates the relationship between inside-out perspective of strategist and financial performance in Vietnamese SMEs.

H7 Organizational creativity mediates the relationship between outside-in perspective of strategist and financial performance in Vietnamese SMEs.

**Fig. 1:**
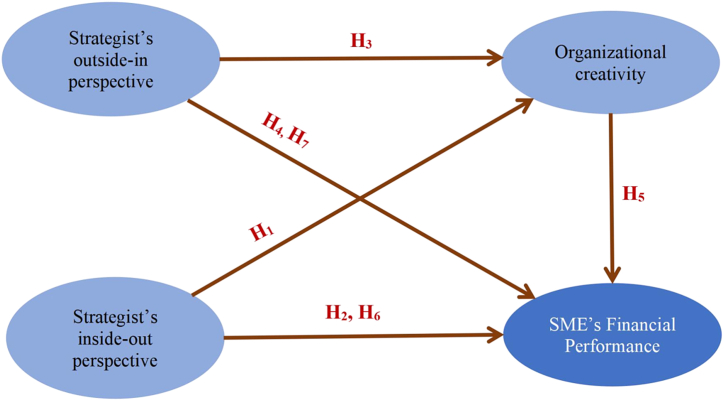
Research model.

The research model is shown in Fig. 1.

## 3 Methods

### 3.1 Measurement scale

The inside-out and outside-in perspectives were each measured by four items, inherited from Ref. [39], and assessed the degree to which the firm's strategy was internally or externally focused. The assessment of organizational creativity involved utilizing five items adapted from Ref. [40] work. Meanwhile, the evaluation of financial performance in this study employed four items that were adapted from Ref. [41] research. All items were rated on a 5-point Likert scale ranging from “strongly disagree” to “strongly agree”, all shown in Table 2.

**Table 2:** Description of construct.

**Variables**	**Items**	**Description**	**Factor loadings**	**References**
Inside-out perspective (IOP)	IOP1	My company sticks to its core technologies and capabilities, seeking new markets where they can be applied.	0.658	Meyer (2007)
	IOP2	My company stays close to the core competencies.	0.667	
	IOP3	The starting point of all strategies is the distinctive capabilities of my company.	0.839	
	IOP4	My company focuses on the market opportunities closest to its core competencies.	0.826	
Outside-in perspective (OIP)	OIP1	If my company jumps at market opportunities, it can always develop the necessary competencies and technologies to match.	0.746	
	OIP2	My company should pursue the best market opportunities, not necessarily the ones closest to the firm's current competencies.	0.647	
	OIP3	My company believes that focusing solely on core competencies can slow its ability to seize market opportunities quickly.	0.736	
	OIP4	My company is always prepared to acquire resources after pursuing a market opportunity.	0.727	
Organizational creativity (CRE)	CRE1	My company has produced many novel and useful ideas (services/products).	0.740	Mikalef & Gupta (2021)
	CRE2	My company fosters an environment that is conducive to its ability to produce novel and useful ideas (services/products).	0.771	
	CRE3	My company spends much time producing novel and useful ideas (services/products).	0.768	
	CRE4	My company considers producing novel and useful ideas (services/products) as an important activity.	0.662	
	CRE5	My company actively produces novel and useful ideas (services/products).	0.698	
Financial performance (FPE)	FPE1	In comparison with competitors, Return on Equity (ROE) of my company increases.	0.791	Saeidi et al. (2015)
	FPE2	In comparison with competitors, Return on Sales (ROS) of my company increases.	0.778	
	FPE3	In comparison with competitors, Return on Assets (ROA) of my company increases.	0.812	
	FPE4	In comparison with competitors, Return on Investment (ROI) of my company increases.	0.740	

### 3.2 Data collection and sample

This research concentrates on Vietnamese SMEs that have operated for at least five years to ensure their survival during the COVID-19 pandemic. The SMEs are defined based on the International Finance Corporation's (2009) criteria, which classify firms with less than 250 employees as SMEs. The research is being conducted in Ho Chi Minh City and Hanoi, Vietnam, as these cities host more than 50% of Vietnamese enterprises (The White book on Vietnam businesses, 2022). The targeted participants for this study are managers at all levels of SMEs, including owners, CEOs, vice directors, management board assistants, and department heads with extensive experience in strategic management.

Prior to commencing data collection, a small yet representative group of respondents (n = 30), including SME managers from various industries and strategic management scholars, underwent a face-to-face interview pretest to assess the questionnaire's construct validity [42]. The pretest aimed to determine whether any questions were too complex to answer due to lengthy sentences, phrasing, or specialized language [43]. The questionnaire was subsequently modified through a back-translation process from English to Vietnamese and vice versa, with the aid of three independent translators and English language instructors [44,45]. The questionnaire was then divided into two sections, with the first and primary part comprising 17 items on the study's theoretical constructs and the second part collecting the respondents' demographic information.

Initially, the sample size was set at 450 randomly chosen participants based on the “10-times rule” for sample size calculation [46]. However, this study determined the initial sample size to be 450 to account for non-response bias. The study period is from December 2022 to February 2023. Two approaches were adopted to recruit the intended number of respondents for the sample. The first approach entailed sending an online survey directly to company emails, with the list of SMEs obtained from databases provided by https://vnr500.com.vn/. The second approach involved distributing self-administered questionnaires through training programs, seminars, and other events organized by SME associations and other agencies in Ho Chi Minh and Hanoi. Informed consent has been obtained from all survey participants to ensure ethical standards for this study examining the relationships between strategic perspectives, organizational creativity, and the financial performance of SMEs in Vietnam. In total, 400 responses were received, resulting in an 88.9% response rate, with 180 online-based responses and 220 paper-based responses. Incomplete responses were eliminated through scanning, leaving 382 completed ones (a response rate of 84.9%).

### 3.3 Analytical procedures

Due to the potential indicator residual variance importance, managing complex constructions and interactions, and handling complex constructs and interactions, PLS-SEM with a composite model is appropriate for the strategists' perspectives, organizational creativity, and SME financial performance study [47,48]. Empirical loadings and error mitigation support this option, which is further supported by non-normal data distribution and model complexity, making PLS-SEM preferred to CB-SEM [49,50] The robustness and statistical power of PLS-SEM also improve the ability to find significant connections [50,51].

The analytical process in this study encompassed several stages, beginning with a collinearity and common method bias test [52,53], followed by an assessment of the measurement model, including the evaluation of aggregate reliability, extracted variance, and the comparison of the square root of the extracted variance with the correlation coefficient. Additionally, discriminant validity was evaluated. Subsequently, the structural model was tested, and the coefficient of determination (R2) was used as the evaluation criterion. Finally, the direct effects of the variables in the model were analyzed using the PLS Bootstrapping technique, with a recommended repeated sample size of 5000, as suggested by Ref. [54]. After that, we used Bootstrapping analysis to determine PLS-SEM means and standard deviations to study organizational creativity's mediating effect on the relationships between two strategists' perspectives (inside-out and outside-in) and financial performance [55]. The coefficient and t-statistics show that exogenous and endogenous latent variables mediate each other.

## 4 Results

### 4.1 Descriptive statistics

According to the data in Table 3, the survey respondents comprised 62.6% male and 37.4% female participants. Most respondents had completed university-level education (73.6%), with 12.3% holding a master's degree and only 2.9% having completed a doctoral degree. Conversely, only a small percentage had completed high school and vocational school (3.7%) or college-level education (7.6%). Regarding their positions within their organizations, the respondents held various roles. The largest group of respondents held the position of head of department (39.3%), followed by company owners (23%), management board assistants (13.9%), vice directors (11.5%), chief executive officers (12.3%), and others (0.9%).

**Table 3:** Descriptive statistics of sample.

Index	n = 382	Intensity (%)
**Gender**		
Male	239	62.6
Female	143	37.4
**Education**		
High School & Vocational School	14	3.7
College level	29	7.6
University level	281	73.6
Master level	47	12.3
Doctoral level	11	2.9
**Position**		
Company owner	88	23.0
Chief Executive Officer	47	12.3
Vice Director	44	11.5
Management Board Assistant	53	13.9
Head of Department	150	39.3

Prior to evaluating the structure model, it is essential to assess the presence of collinearity issues in the inner model. Our study found no evidence of multicollinearity issues as the tolerance values exceeded the threshold of 0.2, and all VIF values remained below the threshold of 5 [56].

Among the samples collected, most respondents were from the trade and service industry, with 211 SMEs represented. This was followed by the production industry, with 99 SMEs, and the real estate industry, with 33 SMEs. A smaller number of respondents were from other industries, with 39 SMEs represented.

### 4.2 Collinearity and common method bias

We conducted several analyses to assess the potential threat of common method bias (CMB). Initially, we performed a Harmon one-factor test [57] on four factors: outside-in perspective, inside-out perspective, organizational creativity, and financial performance. Our findings indicated that CMB was not a significant concern, as all four factors were present, and the greatest covariance explained by one factor was 27.25%. Subsequently, we included a method factor in the PLS model based on [52] recommendations. The method factor's indicators comprised all the principal constructs' indicators. To address the issue of common method bias, we estimated the substantive variance and method variance for each indicator. Based on the results presented in Table 4, the average substantive variance and the average method variance across all indicators were 0.437 and 0.014, respectively, with a ratio of 30:1. Moreover, most of the factor loadings associated with the common method factor were found to be insignificant. Consequently, common method bias has no potential impact on the study findings.

**Table 4:** Common method bias analysis.

		Substantive factor loading		Method factor loading	
**Construct**	**Indicator**	**(R1)**	**R1**^**2**^	**(R2)**	**R2**^**2**^
Inside-out perspective	IOP1	0.677***	0.458329	−0.002	0.000004
	IOP2	0.741***	0.549081	−0.067	0.004489
	IOP3	0.798***	0.636804	0.029	0.000841
	IOP4	0.793***	0.628849	0.029	0.000841
Outside-in perspective	OIP1	0.398***	0.158404	0.341*	0.116281
	OIP2	0.802***	0.643204	−0.132***	0.017424
	OIP3	0.898***	0.806404	−0.152**	0.023104
	OIP4	0.755***	0.570025	−0.018	0.000324
Organizational creativity	CRE1	0.729***	0.531441	0.03	0.0009
	CRE2	0.891***	0.793881	−0.114	0.012996
	CRE3	0.803***	0.644809	−0.029	0.000841
	CRE4	0.548***	0.300304	0.092	0.008464
	CRE5	0.642***	0.412164	0.046	0.002116
Financial performance	FPE1	0.841***	0.707281	−0.044	0.001936
	FPE2	0.9***	0.81	−0.14**	0.0196
	FPE3	0.784***	0.614656	0.029	0.000841
	FPE4	0.588***	0.345744	0.183**	0.033489
Average		0.57	0.437	0.0048	0.0144

**Note(s):** ***p < 0.001, **p < 0.01, *p < 0.05.

### 4.3 Measurement model

To assess the reliability of our measurement scale, we computed both Cronbach's Alpha coefficients and composite reliability. The results in Table 5 indicate that Cronbach's Alpha values for the different constructs ranged from 0.691 (for the outside-in perspective) to 0.789 (for financial performance). The composite reliability values ranged from 0.807 (for outside-in perspective) to 0.862 (for financial performance). These values, which are in close proximity to or greater than the 0.7 threshold [56], demonstrate the structural reliability of the model.

**Table 5:** Assessment results of measurement model.

**Construct**	**Cronbach's Alpha**	**Composite Reliability**	**AVE**
Inside-out perspective	0.743	0.838	0.566
Outside-in perspective	0.691	0.807	0.512
Organizational creativity	0.778	0.850	0.531
Financial performance	0.789	0.862	0.610

Table 5 also displays the Average Variance Extracted (AVE) value for each structure. The value of AVE for all structures in the model is greater than 0.5, indicating convergence of each structure in the model [58]. Furthermore, discriminant validity, the degree to which factors are distinct and not correlated, was assessed. The correlation coefficients between structures were compared with the square root of AVE. Table 6 shows that the square root of all AVEs (ranging from 0.715 to 0.781) is greater than the coefficients in the same column, demonstrating discriminant validity. The Heterotrait-Monotrait (HTMT) correlation ratio was used to evaluate discriminant validity further, as recommended by Ref. [59]. The HTMT values in Table 6 are below the threshold value of 0.90.

**Table 6:** Correlation, AVE and Heterotrait-Monotrait Ratio (HTMT) values.

	**Organizational creativity**	**Financial performance**	**Inside-out perspective**	**Outside-in perspective**
**Organizational creativity**	**0.729**	0.433	0.533	0.518
**Financial performance**	0.351	**0.781**	0.125	0.317
**Inside-out perspective**	0.409	0.047	**0.752**	0.584
**Outside-in perspective**	0.401	0.255	0.446	**0.715**

Notes: Correlations and Heterotrait-Monotrait Ratio are shown below and above the diagonal, respectively; the square roots of AVE are highlighted in bold.

**Table 7:** Hypotheses testing.

**Relationships**	**Path coefficient**	**t-value**	**Results**
**H**_**1**_**.** Inside-out perspective → Organizational creativity	0.287	5.182***	Supported
**H**_**2**_**.** Inside-out perspective → Financial performance	−0.182	2.852**	Supported
**H**_**3**_**.** Outside-in perspective → Organizational creativity	0.273	4.843***	Supported
**H**_**4.**_ Outside-in perspective → Financial performance	0.198	3.259**	Supported
**H**_**5**_**.** organizational creativity → Financial performance	0.346	5.692***	Supported
**H**_**6**_**.** Inside-out perspective → Organizational creativity → Financial performance	0.099	4.024***	Supported
**H**_**7**_**.** Outside-in perspective → Organizational creativity → Financial performance	0.094	3.550***	Supported

Notes: ***p < 0.001, **p < 0.01.

Thus, the results suggest that the measurement model has satisfactory validity and discriminant validity.

### 4.4 Structural model

To test the hypotheses, we ran a bootstrapping procedure with a resampling rate of 5000 [60] to compute the *t*-value. Based on the rule of thumb for two-tailed hypotheses testing, t-value must be over 1.96 (5% significance level); 2.57 (1% significance level) and 3.29 (0.1% significance level) [61,62] and p-values must be smaller than 0.05. Thus, the statistical findings indicate that all seven hypotheses are supported (Table 7).

**Fig. 2:**
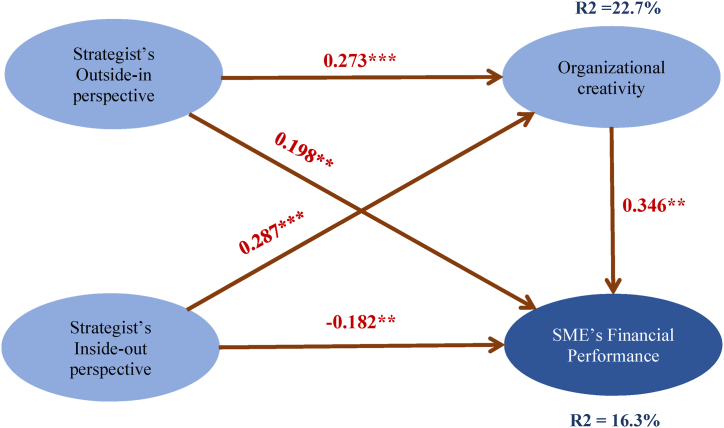
Results of structural model. *Notes.* Paths with **p < 0.01. and ***p < 0.001 are significant.

According to Ref. [62] recommended R2 values for endogenous latent variables, substantial R2 value is 0.26, moderate R2 value is 0.13, and weak R2 value is 0.02. The R2 value for organizational creativity and financial performance is 0.227 and 0.163, respectively (Fig. 2), considered acceptable [62,63]. These findings suggest that the research models have moderate predictive power and are suitable for predicting SMEs' creativity and financial performance.

## 5 Discussion

In this study, we investigate the impact of inside-out and outside-in perspectives of a strategist on organizational creativity and financial performance in Vietnamese SMEs. Our findings reveal that an outside-in perspective positively affects organizational creativity and financial performance, while an inside-out perspective positively affects organizational creativity but negatively impacts financial performance. Our results suggest that the impact of both the inside-out and outside-in perspectives on financial performance is partially mediated by organizational creativity.

The findings are consistent with previous research that has shown the positive impact of an outside-in perspective on financial performance in SMEs [6,32,64]. This emphasizes the importance of understanding and responding to market requirements ahead of competitors, leading to the establishment of strong and enduring customer relationships [64,65]. This approach also emphasizes gathering knowledge about customer needs, competitor capabilities, and market dynamics to create superior value for customers and achieve a favorable market position, ultimately contributing to future returns and competitive advantage [32]**.** Our study provides further evidence to support this perspective and demonstrates that it also positively impacts organizational creativity [6]. The outside-in perspective positively impacts organizational creativity by highlighting customer-centricity, market awareness, external collaborations, risk mitigation, and a culture of innovation, enabling organizations to gain a competitive edge by aligning their creative efforts with customer needs and market trends.

On the other hand, the inside-out oriented company focuses on leveraging internal strengths and capabilities to create value for customers. Rather than starting with the product or service itself, this approach begins with understanding the “why” of the business's existence and its core beliefs. By aligning products and services with the company's essence, businesses can establish a powerful brand identity and appeal to customers who are drawn to the company's mission and values, resulting in a stronger and more loyal customer base [66]. Our findings regarding the inside-out perspective contrast to previous studies that have shown a positive relationship between this perspective and financial performance [6,67]. The inside-out perspective involves an inward-looking approach that emphasizes identifying and leveraging an organization's internal strengths and capabilities. This approach can foster the development of unique and innovative products and services [33]. However, our study shows that this approach negatively impacts financial performance in Vietnamese SMEs. This may be due to SMEs' limited resources and capabilities, which may make it difficult to capitalize on their strengths and may result in a lack of adaptability to changes in the market [64]. In addition, the study supports the positive influence of the inside-out perspective on organizational creativity [6]. Inside-out orientation, with its emphasis on internal strengths, self-awareness, continuous improvement, and fostering a sense of purpose, all contribute to an environment that fosters and encourages creative thinking.

Our study also highlights the importance of organizational creativity in enhancing financial performance in SMEs. This finding is consistent with previous research indicating a positive relationship between creativity and organizational financial performance [68,69]. Creativity allows SMEs to develop innovative products and services that differentiate them from competitors and attract customers [70]. In addition, this approach equips organizations with the ability to respond and adapt to changes in the market, which is essential for ensuring long-term success [69]. Managers can enhance organizational performance and competitiveness by fostering a creative culture within the organization [69,71].

Finally, the study provides evidence that organizational creativity is mediating the relationship between both inside-out and outside-in perspectives and financial performance. This finding underscores the significance of creativity as a critical mechanism through which distinct strategic approaches can affect an organization's financial performance. This is consistent with previous research that has shown the mediating role of creativity in the relationship between different variables and organizational performance [6,72].

## 6 Implications

This study makes significant theoretical contributions. Firstly, this study makes an essential theoretical contribution by exploring the domain of strategic cognition in the context of strategic management for SMEs in Vietnam. While strategic cognition has acquired popularity in recent years [8], there is still a significant knowledge gap concerning how strategists make informed decisions in this context. To address this shortcoming, the study integrates both inside-out and outside-in perspectives within a research model to investigate the role of creativity as a mediator between the two mental perspectives of strategists and SME financial performance. This ground-breaking study sheds light on the complex cognitive processes that guide strategists and their ensuing impact on the financial outcomes of SMEs in Vietnam. Secondly, while RBV and DC have been extensively studied individually [29,73], there is a need to integrate them more cohesively. Our research provided a more holistic view of how firms generate and sustain competitive advantage by combining the static perspective of RBV with the dynamic perspective of DC. By doing so, we aim to shed light on the mechanisms through which firms create and renew valuable resources over time in response to changing environments.

The study's findings have far-reaching managerial implications for managers and policymakers in Vietnam, providing them with invaluable insights to make informed decisions on resource allocation and dynamic capability development for better performance outcomes. The study also emphasizes the criticality of adopting an outside-in perspective, understanding customer needs, and fostering a culture of creativity and innovation to enhance organizational performance and competitiveness. Moreover, the study highlights the crucial role of the cognitive school of thought in strategic management. By comprehending the cognitive processes involved in strategic decision-making, managers can make more effective decisions regarding allocating resources and developing dynamic capabilities [74], helping SMEs in Vietnam adapt to changing market conditions and remain competitive.

## 7 Conclusion, limitations and future studies

In conclusion, our study provides insights into the impact of different strategic perspectives on organizational creativity and financial performance in Vietnamese SMEs. Our findings emphasize the importance of adopting an outside-in perspective and fostering a creative culture within organizations to enhance performance and competitiveness. Our results also highlight the importance of considering organizational creativity as a mediator in the relationship between strategy and financial performance. However, our findings regarding the inside-out perspective contrast previous studies, and further research is needed to explore this relationship in different contexts. Future research could explore the impact of other strategic perspectives on creativity and performance in SMEs and investigate the role of other mediators in the relationship between strategy and performance.

This study has several limitations that need to be addressed in future research. First, the convenience sampling method via social networks may have limited the sample's representativeness, affecting the finding's generalizability. Future studies could adopt probability sampling methods to increase the sample's representativeness. Second, the paper did not consider industry-specific factors that may moderate the relationships between the variables. To address this limitation, further studies could explore how industry-specific factors affect the relationships between inside-out and outside-in perspectives, organizational creativity, and financial performance. Finally, the paper was conducted in a specific cultural context, and cultural factors may have influenced the findings. Future studies could investigate the influence of culture and beliefs on strategic management and examine how they may impact the relationships between inside-out and outside-in perspectives, organizational creativity, and financial performance.

## Funding statement

This research is funded by Vietnam National University HoChiMinh City (VNU-HCM) under grant number B2023-28-03.

## Author contribution statement

Phuong Ngoc-Duy Nguyen: Conceived and designed the experiments; Performed the experiments; Analyzed and interpreted the data; Contributed reagents, materials, analysis tools or data; Wrote the paper.

Khuong Ngoc Mai: Conceived and designed the experiments; Performed the experiments; Analyzed and interpreted the data; Contributed reagents, materials, analysis tools or data; Wrote the paper.

Thu-Hang Le: Conceived and designed the experiments; Performed the experiments; Analyzed and interpreted the data; Wrote the paper.

## Data availability statement

Data will be made available on request.

## Declaration of competing interest

The authors declare the following financial interests/personal relationships which may be considered as potential competing interests:Thu-Hang Le reports financial support was provided by Vietnam National University Ho Chi Minh City.
