# Supplementary material for: Strategic perspectives, creativity, and financial performance in Vietnamese SMEs
Source: Heliyon. 2023 Sep 21;9(9):e20354. doi: 10.1016/j.heliyon.2023.e20354 (PMC10560046; doi:10.1016/j.heliyon.2023.e20354)
Supplement: Multimedia component 1 [file mmc1.pdf]

Strategic Perspectives, Creativity, and Financial Performance in Vietnamese SMEs

Questionnaire

Part 1: Inside-out perspective; Outside-in perspective; Organizational creativity; Financial Performance Constructs

Variables	Items	Full description	References
**Inside-out perspective (IOP)**	IOP1	My company sticks to its core technologies and capabilities, seeking new markets where they can be applied.	Meyer (2007) [1]
	IOP2	My company stays close to the core competencies.	
	IOP3	The starting point of all strategies is the distinctive capabilities of my company.	
	IOP4	My company focuses on the market opportunities closest to its core competencies.	
**Outside-in perspective (OIP)**	OIP1	If my company jumps at market opportunities, it can always develop the necessary competencies and technologies to match.	
	OIP2	My company should pursue the best market opportunities, not necessarily the ones closest to the firm's current competencies.	
	OIP3	My company believes that focusing solely on core competencies can slow its ability to seize market opportunities quickly.	
	OIP4	My company is always prepared to acquire resources after pursuing a market opportunity.	
**Organizational creativity (CRE)**	CRE1	My company has produced many novel and useful ideas (services/products).	Mikalef & Gupta (2021) [2]
	CRE2	My company fosters an environment that is conducive to its ability to produce novel and useful ideas (services/products).	
	CRE3	My company spends much time producing novel and useful ideas (services/products).	
	CRE4	My company considers producing novel and useful ideas (services/products) as an important activity.	
	CRE5	My company actively produces novel and useful ideas (services/products).	
**Financial performance (FPE)**	FPE1	In comparison with competitors, Return on Equity (ROE) of my company increases.	Saeidi et al. (2015) [3]
	FPE2	In comparison with competitors, Return on Sales (ROS) of my company increases.	
	FPE3	In comparison with competitors, Return on Assets (ROA) of my company increases.	
	FPE4	In comparison with competitors, Return on Investment (ROI) of my company increases.	

Part 2: Demographic information

1. My gender is
☐ Male
☐ Female

2. My educational level is:
☐ High School & Vocational School
☐ College level
☐ University level
☐ Master level
☐ Doctoral level

3. My position in the company is:
☐ Company owner
☐ Chief Executive Officer
☐ Vice Director
☐ Management Board Assistant
☐ Head of Department

References

[1] Meyer R. Mapping the mind of the strategist: a quantitative methodology for measuring the strategic beliefs of executives. 2007.
[2] Mikalef P, Gupta M. Artificial intelligence capability: Conceptualization, measurement calibration, and empirical study on its impact on organizational creativity and firm performance. Information & Management 2021;58:103434.
[3] Saeidi SP, Sofian S, Saeidi P, Saeidi SP, Saaeidi SA. How does corporate social responsibility contribute to firm financial performance? The mediating role of competitive advantage, reputation, and customer satisfaction. J Bus Res 2015;68:341–50.
